# Supplementary material for: Identification of photoperiod-induced specific miRNAs in the adrenal glands of Sunite sheep (Ovis aries)
Source: Front Vet Sci. 2022 Jul 22;9:888207. doi: 10.3389/fvets.2022.888207 (PMC9354845; doi:10.3389/fvets.2022.888207)
Supplement: Supplementary file 1 [file Data_Sheet_1.ZIP › Supplementary file/miRDeep2 file.zip!/miRDeep2 file/Know_Structure/SP42A2/oar-mir-181a-1.pdf]

miRBase precursor	: oar-mir-181a-1
Total read count	: 17715
oar-miR-181a read count	: 17635
remaining reads	: 80

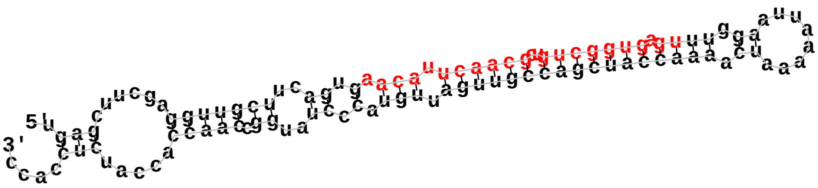

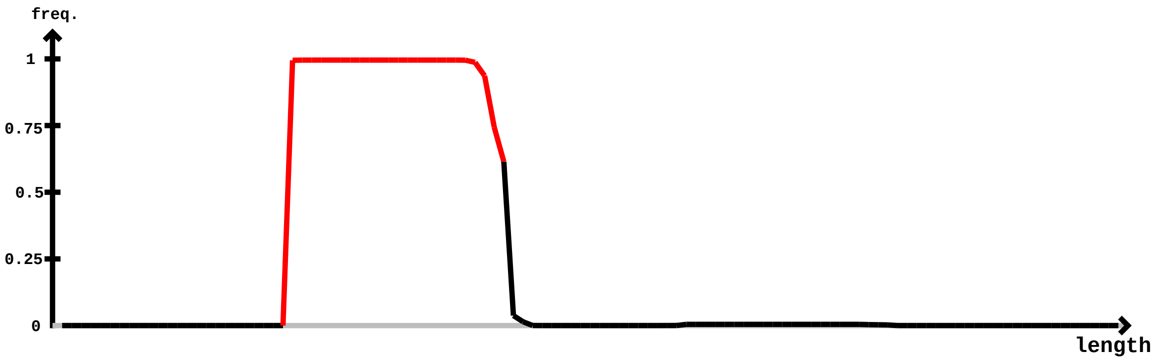


```
                              oar-miR-181a
5'- ugagcuucgagguugcuucagugaacauucaacgcugucggugaguuuggaauuaaaaaucaaaaccaucgaccguugauuguacccuauggccaaccaccaucuccacc   -3'    exp
    .(((......((((((..((.(.(((.((((((..(((((((.((((.((........)).)))))))))))))))))).))).).))..)).))))).....)))....   reads      mm         sample
    .......................aacauucaacgcugucgg.....................................................................   6          0          seq
    .......................aacauucaacgcugucggu....................................................................   145        0          seq
    .......................aacauucaacgcugucggug...................................................................   894        0          seq
    .......................aacauucaacgcugucgguga..................................................................   3410       0          seq
    .......................aacauucaacgcugucggugag.................................................................   2294       0          seq
    .......................aacauucaacgcugucggugagu................................................................   10210      0          seq
    .......................aacauucaacgcugucggugaguu...............................................................   409        0          seq
    .......................aacauucaacgcugucggugaguuu..............................................................   250        0          seq
    .......................aacauucaacgcugucggugaguuug.............................................................   3          0          seq
    ........................acauucaacgcugucgguga..................................................................   1          0          seq
    ........................acauucaacgcugucggugag.................................................................   1          0          seq
    ........................acauucaacgcugucggugagu................................................................   10         0          seq
    ........................acauucaacgcugucggugaguuu..............................................................   1          0          seq
    ..........................auucaacgcugucggugaguu...............................................................   1          0          seq
    ..............................................................aaaccaucgaccguugauugu...........................   1          0          seq
    ...............................................................aaccaucgaccguugaGu.............................   1          1          seq
    ...............................................................aaccaucgaccguugauugu...........................   1          0          seq
    ...............................................................aaccaucgaccguugauuguacc........................   1          0          seq
    ................................................................accaucgaccguugauugu...........................   13         0          seq
    ................................................................accaucgaccguugauugua..........................   11         0          seq
    ................................................................accaucgaccguugauuguac.........................   10         0          seq
    ................................................................accaucgaccguugauuguacc........................   41         0          seq
    .................................................................ccaucgaccguugauuguacc........................   1          0          seq
```
